# LCR-modules: a collection of workflows for cancer genome analysis

**DOI:** 10.1093/bioinformatics/btag366

**Published:** 2026-06-10

**Authors:** Kostiantyn Dreval, Laura K Hilton, Bruno M Grande, Giuliano Banco, Krysta M Coyle, Manuela Cruz, Sierra Gillis, Luke Klossok, Prasath Pararajalingam, Christopher K Rushton, Haya Shaalan, Nicole Thomas, Helena Winata, Jasper Wong, Jacky Yiu, Christian Steidl, David W Scott, Ryan D Morin

**Affiliations:** Department of Molecular Biology and Biochemistry, Simon Fraser University, Burnaby, BC, V5A 1S6, Canada; Basic and Translational Research, BC Cancer Research Institute, Vancouver, BC, V5Z 1L3, Canada; Centre for Lymphoid Cancer, BC Cancer Research Institute, Vancouver, BC, V5Z 1L3, Canada; Department of Molecular Biology and Biochemistry, Simon Fraser University, Burnaby, BC, V5A 1S6, Canada; Department of Molecular Biology and Biochemistry, Simon Fraser University, Burnaby, BC, V5A 1S6, Canada; Department of Molecular Biology and Biochemistry, Simon Fraser University, Burnaby, BC, V5A 1S6, Canada; Department of Molecular Biology and Biochemistry, Simon Fraser University, Burnaby, BC, V5A 1S6, Canada; Basic and Translational Research, BC Cancer Research Institute, Vancouver, BC, V5Z 1L3, Canada; Centre for Lymphoid Cancer, BC Cancer Research Institute, Vancouver, BC, V5Z 1L3, Canada; Department of Molecular Biology and Biochemistry, Simon Fraser University, Burnaby, BC, V5A 1S6, Canada; Department of Molecular Biology and Biochemistry, Simon Fraser University, Burnaby, BC, V5A 1S6, Canada; Department of Molecular Biology and Biochemistry, Simon Fraser University, Burnaby, BC, V5A 1S6, Canada; Department of Molecular Biology and Biochemistry, Simon Fraser University, Burnaby, BC, V5A 1S6, Canada; Department of Molecular Biology and Biochemistry, Simon Fraser University, Burnaby, BC, V5A 1S6, Canada; Department of Molecular Biology and Biochemistry, Simon Fraser University, Burnaby, BC, V5A 1S6, Canada; Centre for Lymphoid Cancer, BC Cancer Research Institute, Vancouver, BC, V5Z 1L3, Canada; Department of Molecular Biology and Biochemistry, Simon Fraser University, Burnaby, BC, V5A 1S6, Canada; Centre for Lymphoid Cancer, BC Cancer Research Institute, Vancouver, BC, V5Z 1L3, Canada; Centre for Lymphoid Cancer, BC Cancer Research Institute, Vancouver, BC, V5Z 1L3, Canada; Division of Medical Oncology, Department of Medicine, University of British Columbia, Vancouver, BC, V5Z 4E6, Canada; Department of Molecular Biology and Biochemistry, Simon Fraser University, Burnaby, BC, V5A 1S6, Canada; Basic and Translational Research, BC Cancer Research Institute, Vancouver, BC, V5Z 1L3, Canada; Centre for Lymphoid Cancer, BC Cancer Research Institute, Vancouver, BC, V5Z 1L3, Canada

## Abstract

**Motivation:**

The surge of genomic data from advanced sequencing technologies is outpacing current analytical pipelines. We introduce LCR-modules, an open-source suite of bioinformatics tools designed for flexible and automated cancer genome data analysis. LCR-modules enables reproducible analysis of diverse cancer genomics data at scale. The suite comprises 49 Snakemake-based workflows organized into three levels, facilitating tasks from low-level quality control to complex cohort-level analyses. LCR-modules supports various sequencing types and integrates pipelines such as mutation calling, expression quantification, and cohort-level aggregation, ensuring flexibility and reproducibility. LCR-modules represents a significant advancement in genomic data analysis, reducing barriers in reproducibility and scalability and has already been applied to a combination of exomes and genomes from over 10 800 samples.

**Availability:**

No new data were generated in support of this research. The source code for the LCR-modules is openly available at https://github.com/LCR-BCCRC/lcr-modules.

## 1 Introduction

High-throughput sequencing has been transformational to genetic research and the study of cancer (Liu *et al.* 2019, Satam *et al.* 2023, Mustafa 2024). Large consortia, such as The Cancer Genome Atlas (Weinstein *et al.* 2013) and ICGC (Hübschmann *et al.* 2021), and collective work from thousands of teams globally have generated vast amounts of sequencing data from cancer biopsies. Generating high-quality simple somatic mutation and copy number alteration calls involves a complex collection of algorithms (Ulrich 2017, Cremin *et al.* 2022). Bioinformatics workflows commonly rely on a variety of programming languages, which are cumbersome to maintain and difficult to scale as analyses grow in scope and complexity (Dash *et al.* 2019, Wratten *et al.* 2021). Hence, despite the opportunity afforded by a wealth of cancer sequencing data to reveal novel molecular insights, the ability to rapidly process data in a reproducible manner creates a bottleneck.

For research to keep pace with the influx of data, pipelines to automate routine analytical steps are essential. Ideally, analytical pipelines balance versatility, tuneability, and ease of installation and maintenance while minimizing risks of errors. Modularization and version control facilitate the consistent application of analytical best practices and allow deployment of new features and improvements across projects (Davis-Turak *et al.* 2017, Houssein *et al.* 2023). Reliance on modular components helps promote consistency and reproducibility while reducing the amount of lead time in setting up a new analytical pipeline.

To support the routine analyses of short-read exome, panel-based, transcriptome, whole genome sequencing (WGS) and long-read sequencing, we developed a suite of tools collectively named Lymphoma Cancer Research modules (LCR-modules). This toolkit has been used by our team to discover drivers and molecular features in cancer, specifically focusing on lymphoid neoplasms, and perform quantitative comparisons between disease entities (Bal *et al.* 2022, Dreval *et al.* 2022, 2023, Hilton *et al.* 2023, Thomas *et al.* 2023, 2025, Sahasrabuddhe *et al.* 2025). The collection of LCR-modules includes open-source and custom bioinformatics tools utilizing the Snakemake (Mölder *et al.* 2021) workflow management system, and modules for data quality control, discovery and annotation of common mutation types, analysis of B-cell receptor repertoires, and discovery of loci affected by aberrant somatic hypermutation. Individual modules are configured to provide an automated, scalable, and reproducible workflow that runs each step as dictated by the availability of new data. LCR-modules is actively maintained and openly available on GitHub.

## 2 Results

### 2.1 LCR-modules overview

**Figure 1:**
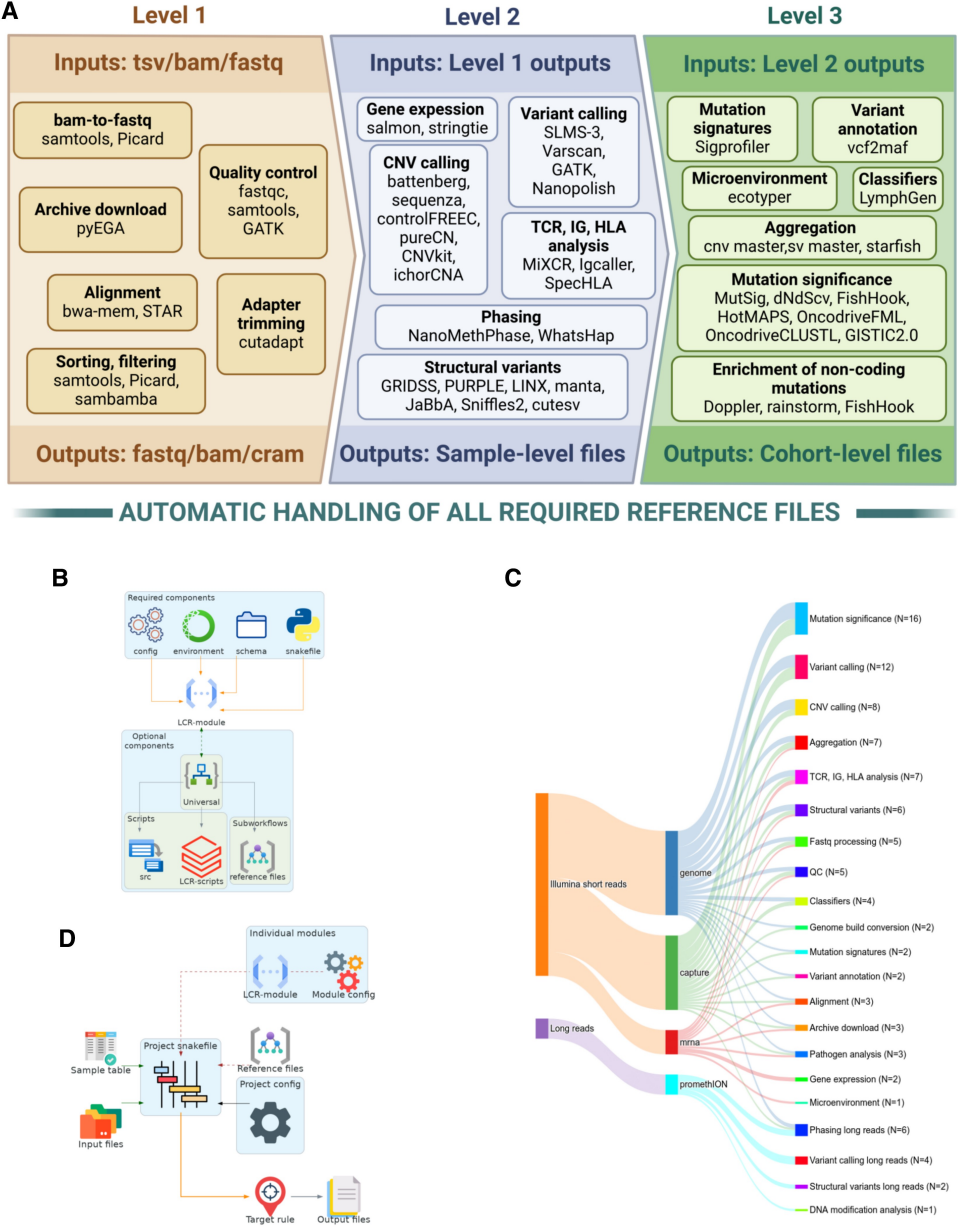
LCR-modules overview. (A) Schematic representation of modules as organized into different levels according to the complexity of performed analyses. Only representative individual modules are shown. The full list of modules is available at https://github.com/LCR-BCCRC/lcr-modules/tree/master/modules. (B) The general structure of the main components of individual modules. (C) Sankey plot showing the relationship between supported sequencing type and generalized function of each module. The number in parentheses illustrates the number of supported sequencing types and individual modules for the specific type of analysis. (D) Schematic representation of the main components in a project workflow organized with LCR-modules.

LCR-modules is a collection of modules that form the basis of workflows to perform scalable and reproducible genomic analyses. The individual modules can be logically divided into three main categories representing the processing of raw, sample-level or cohort-level data (Fig. 1A). The outputs of the modules of each previous level flow, individually or in combination, to modules at the subsequent level.

An important feature of the modules at all levels includes a specifically designed set of processes to handle required reference files automatically with minimal effort from the user. These reference processes handle the download of fasta files for majority of the most common human genome builds and bed files of target regions for commonly used exome panels. While a wide range of genome builds is available to the user out-of-the box, the reference files subworkflow also provides flexibility to add custom reference files or capture-based panels.

Level 1 modules perform low-level tasks such as adapter trimming, quality control, and alignment of sequencing files, and obtaining data from repositories such as the European Genome-phenome Archive (EGA). These modules also perform gene expression analyses, including alignment using STAR (Dobin *et al.* 2013) and calculating mRNA abundance using salmon (Patro *et al.* 2017). Level 2 modules perform routine tasks for cancer analysis, such as detecting and annotating simple somatic mutations, copy-number alterations, and structural variations. Next, the level 3 modules perform analyses that rely on cohort-level aggregation. The cohorts and data sets can be flexibly defined based on different clinical characteristics through a set of configuration files. The modules at this level operate on the outputs of level 2 modules and perform tasks such as aggregation of individual files into cohort-level merges. Example workflows include analyses of mutation signatures, identification of significantly mutated genes, and sample classification into genetic subgroups.

Each of the modules has required and optional components (Fig. 1B). Required components include: the Snakefile (rules), schema (definition of wildcards), conda environment (dependencies), and configuration file (module config). The module config defines the input/output paths, tool parameters, computational resource requirements, supported sequencing types (seq_type), and toggles for handling data with or without a matched normal. Optional components include a reference subworkflow (automated reference assets with user overrides) and shared custom scripts [provided within src directory or via the companion LCR-scripts (https://github.com/LCR-BCCRC/lcr-scripts) repository]. This structure standardizes behavior, recycles common resources, and minimizes code duplication. To improve handling of routine tasks—like subsetting sample table, generating tumor-normal pairs, creating directory structure for the module run, symlinking files, etc.—LCR-modules utilize a custom Python-based oncopipe module (https://pypi.org/project/oncopipe/), developed by our group.

The current collection includes 50 workflows designed to support both short-read Illumina and long-read PromethION sequencing data. The scope of supported sequencing types (seq_type) currently includes genome, capture, mrna, and long-read sequencing data (Oxford Nanopore). Most of the modules support more than one seq_type, often with automatic configurations tailored to the data (Fig. 1C). WGS (genome seq_type) has the highest number of supporting modules, and somatic variant calling and identification of significantly mutated genes are the analysis types with the highest representation of the developed modules.

### 2.2 Assembling modules into pipelines

Any individual modules with compatible inputs or outputs can be combined to facilitate multi-step genomic analysis workflow. Built using Snakemake (Mölder *et al.* 2021), modules can be directly imported into a project Snakefile, exposing their individual rules to the master (project) workflow (Fig. 1D). To establish a project workflow, each of the modules with its associated config constitutes an individual step, and the individual jobs that will be executed are dictated by the contents of the sample table, the presence of compatible input files, and the target rule definitions. The default configuration values of each of the modules can be replaced with the values specified in the project-level config. Such architecture allows the entire project to be concise, easy to maintain, scalable, and reproducible.

The LCR-modules repository provides a demo project where individual modules are organized into a representative workflow based on their sequencing type. The demo project illustrates the concept of the project for users while also ensuring the maintenance of functionality for the entire codebase with every new update. In addition, the demo contains custom wrapper scripts to launch the workflows as a dry run with local execution or with cluster execution using schedulers and workload managers such as Sun Grid Engine and Slurm (Yoo *et al.* 2003).

One example of such workflow is the simple somatic variant calling pipeline in the demo project (SLMS-3; https://github.com/LCR-BCCRC/lcr-modules/tree/master/modules/slms_3). Within both capture- and WGS-based pipelines (https://github.com/LCR-BCCRC/lcr-modules/tree/master/demo), SLMS-3 independently runs mutect2 (Benjamin *et al.* 2019), strelka2 (Kim *et al.* 2018), SAGE (https://github.com/hartwigmedical/hmftools/tree/master/sage), LoFreq (Wilm *et al.* 2012), and manta (Chen *et al.* 2016) variant callers. Starfish (https://github.com/dancooke/starfish) is used to intersect the resulting variant calls from each individual tool such that only variants supported by three or more variant callers are retained. Custom scripts, customized versions of individual callers, and all other required components of SLMS-3 are directly supplied with LCR-modules and pre-configured for the user.

Reproducibility in cancer genomics depends on clear, inspectable pipelines, yet pipeline management is challenged by large datasets, variable genome references and assays, heterogeneous tools, and scaling needs. LCR-modules address each of these issues with modular Snakemake workflows, pinned conda environments, and a reference subworkflow that automates genome assets. Declarative configs (not ad hoc scripts) control inputs, resources, reference files, and seq_type handling, reducing developer effort while preserving transparency. Combined with reusable utilities and an included demo for end-to-end testing, this design promotes consistent results across cohorts and infrastructure, and delivers accuracy on FFPE samples while scaling to large studies. To date, LCR-modules has powered analyses in numerous publications (Dreval *et al.* 2022, 2023, Hilton *et al.* 2023, Thomas *et al.* 2023, 2025, Sahasrabuddhe *et al.* 2025) to perform sequencing sample QC, identify somatic mutations, significantly mutated genes, genetic subgroup assignment—and has processed more than 2060 whole genomes, 4500 exomes, and 4130 RNA-seq datasets, highlighting both its reliability and broad applicability.

## Data Availability

No new data were generated in support of this research. The source code for the LCR-modules is openly available at https://github.com/LCR-BCCRC/lcr-modules.
